# Mechanisms Underlying the Visual Benefit of Cell Transplantation for the Treatment of Retinal Degenerations

**DOI:** 10.3390/ijms20030557

**Published:** 2019-01-28

**Authors:** Thierry Léveillard, Laurence Klipfel

**Affiliations:** Department of Genetics, Sorbonne Université, INSERM, CNRS, Institut de la Vision, 17 rue Moreau, F-75012 Paris, France; laurence.klipfel@inserm.fr

**Keywords:** photoreceptors, retinal pigmented epithelium, retinitis pigmentosa, age-related macular degeneration, cytoplasmic material transfer, induced-pluripotent stem cells

## Abstract

The transplantation of retinal cells has been studied in animals to establish proof of its potential benefit for the treatment of blinding diseases. Photoreceptor precursors have been grafted in animal models of Mendelian-inherited retinal degenerations, and retinal pigmented epithelial cells have been used to restore visual function in animal models of age-related macular degeneration (AMD) and recently in patients. Cell therapy over corrective gene therapy in inherited retinal degeneration can overcome the genetic heterogeneity by providing one treatment for all genetic forms of the diseases. In AMD, the existence of multiple risk alleles precludes a priori the use of corrective gene therapy. Mechanistically, the experiments of photoreceptor precursor transplantation reveal the importance of cytoplasmic material exchange between the grafted cells and the host cells for functional rescue, an unsuspected mechanism and novel concept. For transplantation of retinal pigmented epithelial cells, the mechanisms behind the therapeutic benefit are only partially understood, and clinical trials are ongoing. The fascinating studies that describe the development of methodologies to produce cells to be grafted and demonstrate the functional benefit for vision are reviewed.

## 1. Introduction 

### 1.1. Expectation of Cells Transplantation for the Treatment of Retinal Degenerations

Retinal degenerations are defined by two distinct genetic entities. Inherited retinal degenerations are a monogenic but genetically heterogeneous group of diseases caused by inherited or de novo mutations, found in over 260 disease genes identified so far [1]. Age-related macular degeneration (AMD) is a late-onset multifactorial disease resulting from the interaction between multiple genetic susceptibility alleles and environmental factors [2]. The most frequent form of inherited retinal degeneration, retinitis pigmentosa, affects 2 million people worldwide [3]. Retinitis pigmentosa is characterized by a progression from night blindness due to rod photoreceptor death, followed by the dysfunction and degeneration of cones concentrated in the fovea at the center of the retina, leading to a broad therapeutic strategy applicable to many retinal degenerations [4]. Optogenetic strategies have also been evaluated for vision restoration. Originally, an adeno-associated virus (AAV) was used to deliver in the eye a microbial opsin, channelrhodopsin-2 from *Chlamydomonas reinhardtii*, which was followed by successful attempts using other microbial opsins as well as human rhodopsin in order to better adapt the excitation spectra of optogenetic tools to human vision rescue and to ameliorate the transgene expression [5,6,7,8]. Albeit independent of cell transplantation, optogenetics strategies share the same aim as photoreceptor transplantation—to restore the photosensitivity of blind retinas [9]. Cell replacement could theoretically restore vision in advanced stages of disease with severe cell loss.

AMD affects primarily central vision that is sustained by cone photoreceptors. In primates, the macula at the center of the retina contains a region enriched in cone photoreceptors, the fovea. AMD is the leading cause of blindness in industrialized countries. The estimated prevalence of advanced AMD is only of 0.2% at ages 55 to 64 years old, but increases to 13% in those older than 85 [10]. Age and a positive family history of AMD are the two strongest risk factors for AMD. As the average age of the population in industrialized countries increases, so will the incidence of AMD. Genome wide association studies (GWAS) have led to the identification of AMD susceptibility genes pointing out the implication of the innate immune response in AMD [11,12]. Apart from anti-vascular endothelial growth factor (VEGF) medication that limits the progression of choroid neovascularization, a clinical form of AMD that is less frequent than the predominant form, geographic atrophy, there is no cure for this disease [13]. In AMD, one of the prominent accepted hypotheses proposes that central vision loss results from a dysfunction of the functional interaction between cone photoreceptors and the retinal pigmented epithelium (RPE) [14]. Since gene therapy is currently not a sustainable option for this aging disease, numerous studies have promoted transplantation of RPE cells as a rational therapeutic approach for AMD [15].

Recently, the field of transplantation as a therapy for blinding diseases has received increasing attention. Excellent reviews cover extensively the field of transplantation as a therapy for blinding diseases (for reviews see [16,17,18]). The present review will focus on illustrative examples that greatly advance the current understanding of the mechanisms involved in the benefit of cell transplantation for vision.

### 1.2. Overview of Cell Transplantation

**Figure 1:**
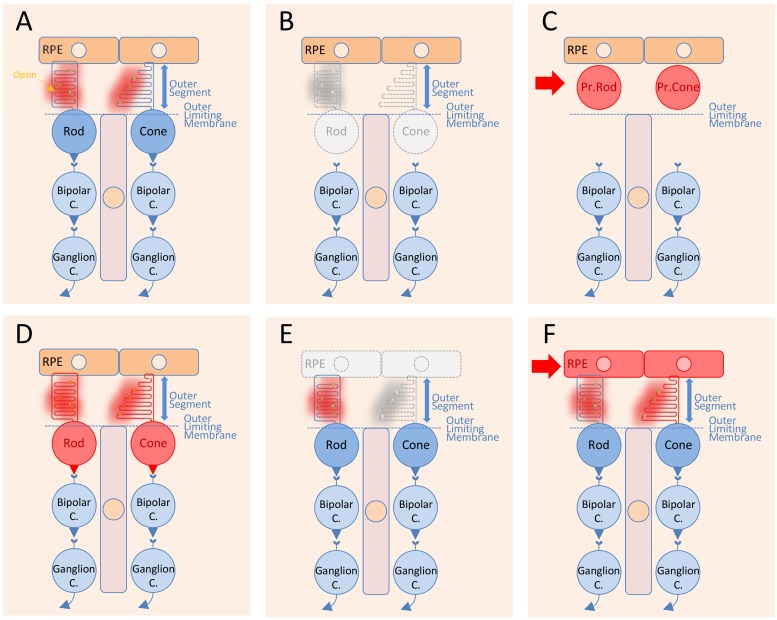
Illustration of the general principle of retinal cell transplantation. (**A**) Healthy retina and retinal pigmented epithelial cells. (**B**) Loss of photoreceptor cells in Mendelian inherited retinal degenerations. (**C**) Transplantation of genetically normal photoreceptor precursors (Pr. Rod or Pr. Cone) in the subretinal space of a photoreceptor-less retina. (**D**) Restoration of the synaptic connectivity of the transplanted photoreceptor precursors with the bipolar cells of the host. (**E**) Dysfunctional retinal pigmented epithelial cells leading to cone dysfunction and cone outer segment shortening at the level of the fovea in age-related macular degeneration. (**F**) Transplantation of healthy and mature retinal pigmented epithelial cells replacing the defective one from the host and restoration of cone function.

The transplantation of RPE cells is distinct from that of photoreceptors for several reasons. The subretinal space is limited by the apical side of the RPE in contact with photoreceptor outer segments and the outer limiting membrane (Figure 1A). In retinitis pigmentosa, rods degenerate through a direct effect of the mutation, while the cones degenerate at least partly in a non-cell autonomous manner (Figure 1B). Photoreceptor precursors, either rods or cones, are transplanted in the subretinal space and stay confined to this position by the outer limiting membrane (Figure 1C). The objective of photoreceptor transplantation is to establish synaptic connectivity between the transplanted cells during their maturation in situ (Figure 1D). In AMD, the cellular defect is likely localized within the RPE cells and results in the macula in the dysfunction of cones centered at the fovea [19] (Figure 1E). Here, transplantation of RPE cells replaces the damaged RPE cells from the host and restores cone function (Figure 1F). Transplantation surgery creates a retinal detachment, a separation of the RPE from the photoreceptor outer segments. The grafted RPE cells are in direct contact with the photoreceptor outer segments without having to cross the outer limiting membrane and have the possibility to reattach to the photoreceptor outer segments. This phenomenon happens naturally in adults, without the requirement of any specific differentiation stage [20]. Consequently, while photoreceptor transplantation focus on immature cells, RPE transplantation uses mature RPE cells.

## 2. Transplantation of Photoreceptors in Inherited Retinal Degenerations

### 2.1. Principles and Limitations

Cell transplantation is a tempting therapeutic approach to replace lost neurons in the central nervous system [21] (Figure 1A–D). The retina is accessible to surgery and convenient for cell delivery. The organization of retinal neurons in spatially distinct layers of distinct cells offers conditions in which any missing neurons could be replaced by cell transplantation. Compared to gene therapy, replacement of missing rods and cones could theoretically provide a therapeutic approach to treat retinitis pigmentosa at late stages, independently of the causal mutation in any of the 64 genes known today. The preservation of the inner retina in retinitis pigmentosa patients, which comprises interneurons and ganglion cells that relay the electrophysiological signal triggered by light at the level of photoreceptors, suggests that transplantation of photoreceptors could prevent blindness [22]. At the advanced stage of the disease, when the patient’s outer nuclear layer (ONL) made of 95% of rods and only 5% of cones has completely degenerated, transplantation of photoreceptors from a healthy donor might regenerate this layer and restore visual function. The first challenge was to find experimental conditions in animal models of retinitis pigmentosa where the integrated photoreceptors could reconnect with neurons of the remaining inner retina to provide the proof-of-concept that photoreceptor transplantation is a beneficial therapy approach [23]. This initial question, which is far from being resolved, is whether the transplanted photoreceptors could make synapses with adult host inner retinal cells [24]. Early attempts using adult photoreceptors failed, and researchers hypothesized the existence of a permissive window during retinal development during which immature photoreceptors could recapitulate the development program that leads to synapse formation with the inner retinal cells. Brain wiring during development is mediated by a series of precisely orchestrated and specific developmental events regulated by ordered molecular mechanisms [25]. One cue came from the transcriptional control of differentiation of photoreceptors from post-mitotic photoreceptor precursors. The current model for the cone/rod switch involves the expression of neural retina-specific leucine zipper (*Nrl*) gene in only a subset of photoreceptor precursors, which become rods [26,27]. Transplanted cells originating from the developing retina, at a time coincident with the peak of rod genesis identified through the onset of *Nrl* expression, can integrate into the degenerating retina of a mouse model of retinitis pigmentosa [28]. These transplanted cells differentiate into rod photoreceptors and form synaptic connections to improve visual function [29]. Integration of the transplanted photoreceptor precursors in the host retina was observed in six murine models of inherited photoreceptor degeneration, but with differences attributed to the gene defect but not to the severity of the disease [30]. The integration into the host ONL of the transplanted cells was evidenced by their visualization through a green fluorescent protein (GFP) transgene reporter. Unfortunately, the corresponding stage of development in human is during the second trimester; consequently, the translation of this approach to treat retinitis pigmentosa patients is currently not medically feasible [31].

Induced-pluripotent stem cell (iPSC) generation from human skin biopsy, in specific culture conditions, forms retinal organoids that recapitulate human retinal development [24]. iPSCs currently represent the most accessible source of cells for transplantation, as they are renewable and can give rise to all somatic cell types [32,33,34]. This in vitro system also permits ensuring safety, since transplanted cells should not contain mitotic cells or residual undifferentiated precursor cells that could be tumorigenic [35,36]. The therapeutic benefit of retinal organoid transplantation has been demonstrated in primates, but the existence of synaptic connection between cells of the organoid indicates that the translation to the clinic will be rationalized by the development of robust strategies to isolate and purify photoreceptors from retinal organoids that contain many other retinal cells [37,38]. In that context, patient-derived iPSCs may be the optimal clinical setting since they bypass the controversial use of embryonic or fetal tissue, and they offer the best possible immunologic match to the patient [39]. Before transplantation, the genetic defect at the origin of the retinal disease must be repaired. Clustered regularly interspaced short palindromic repeats (CRISPR)-Cas9 technology can edit any human loci by inducing double-strand breaks in the gene of interest. Non-homologous end joining then introduces insertions or deletions to inactivate the mutated genes in the case of gain of function mutations or using template-mediated homology-directed repair to correct mutations for recessive genes or dominant genes resulting in haploinsufficiency [22].

### 2.2. Unsuspected Effect

Transplantation of large numbers of post-mitotic rod precursors or iPSCs improves visual function in various murine models of retinitis pigmentosa [40]. However, a detailed analysis of the phenomenon revealed that functional recovery might result from transferring of cytoplasmic material from transplanted rods to remaining host photoreceptors, rather than through integration into the recipient ONL followed by de novo synapse formation with the interneurons of the inner retina [4]. This intercellular material exchange accounts for the majority of GFP-labeled cells within the ONL of the host retina and questions the cellular mechanisms of rescue. The transplantation of photoreceptor precursors isolated from mice carrying a disruption of genes mutated in the host retina should clarify the importance of this phenomenon in the functional benefit observed after transplantation, but surprisingly such an experiment has not yet been reported. The exchange of cytoplasmic material is restricted to photoreceptor–photoreceptor or Müller-cell–photoreceptor interactions and not to other cells in the retina [41]. The mechanisms by which this occurs are presently unknown but do not result from fusions of cells or nuclei between the transplanted photoreceptors, since no GFP-positive cell integrated into the host retina with a male nucleus could be detected after transplantation of male photoreceptor cells into female hosts [42]. It also does not result from the release and uptake of free GFP protein from the interphotoreceptor matrix, extracellular space between the photoreceptor outer segments, and the RPE. Many distinct cytoplasmic RNAs and/or proteins are exchanged between grafted rod precursors and adult host photoreceptors, and it seems that the amount of material exchanged is sufficient to confer functionality of the mutated recipient cells. In that scenario, material transfer permits the restoration of the mutated host rod function and the transplanted cells will only be vehicles. The benefit of the transplantation will not result from the integration and connection of the cells transplanted but from the restoration of the function of the host photoreceptors by cytoplasmic exchange. The exchange of cytoplasmic material occurs preferentially in regions of the retina with disruption of the outer limiting membrane, but not exclusively. The outer limiting membrane is made of apical processes of Müller glial cells attached together and to the inner segments of photoreceptors, and provides a semi-permeable barrier through adherent junctions that have an adhesive role to maintain tissue integrity against mechanical stress [43]. Intuitively, to reach the ONL, the cytoplasmic material from transplanted cells that is located in the subretinal space must either cross the outer limiting membrane, or alternatively transit through Müller cells by entering the apical processes of Müller glial cells and exiting in the ONL on the other side of the outer limiting membrane. The observation of Müller cells that are GFP-positive is an argument in favor of the second hypothetical mechanism but does not resolve the question [44]. Probably distinct from this phenomenon, vision restoration was reported after the de novo Müller cell-derived genesis of rod photoreceptors in mammalian retinas [45]. It has been demonstrated already that surgery trauma resulting from photoreceptor transplantation activates Müller cells that increase, in response to the injury, the expression of glial fibrillary acidic protein (GFAP) [46]. Retinal microglial cells move from the inner retina to the area of surgical damage. These resident microglial cells and invading innate immune cells participate in the response to surgical damage to preserve tissue, and they can also promote neuroinflammation [47]. Inflammation may damage the integrity of the outer limiting membrane facilitating the transfer of cytoplasmic material from transplanted cells into the subretinal space to the ONL, as observed when delivering mesencephalic astrocyte-derived neurotrophic factor (MANF/ARMET), a factor that activates innate immune cells [48]. GFP-labeled-donor cells displaying extending processes toward the host retina after transplantation were interpreted as donor cells migrating toward the recipient ONL [4]. This observation likely corresponds to the capture of the cytoplasmic material exchange process.

Cone regeneration will have a much greater medical impact than rod regeneration since cones are responsible for the detection of color, daylight vision, and high visual acuity [49]. The dual retina of vertebrates contains rod and cone photoreceptors in proportions that vary extensively among species. The biology of photoreceptors was originally studied in species with cones and rods in equal proportions, but the work on transplantation was performed in rodents, which, like most other mammal species, have a large proportion of rods due to an evolutionary phenomenon called nocturnal bottleneck [50]. For technical reasons, earlier studies were conducted by transplanting rod photoreceptors, since cones represent only 3% of mouse retinal photoreceptors. The development of iPSCs could overcome this limitation by driving precursor cells to cone cell-fate by ectopic expression of morphogens as the transcription factors retinoid-acid receptor gamma (RXRG) and thyroid hormone receptor beta isoform 2 (THRB2) to force their differentiation to medium wave cones [51,52]. Even with favorable conditions that offer the possibility of driving the differentiation of iPSCs toward cone cell-fate in vitro, it is still challenging to achieve high levels of cone enrichment for transplantation because of the low proportion of cones in the starting material. Transcriptome analysis is used as a guide to measure cone identity of iPSC-derived cells in comparison to fetal cones to obtain cones for transplantation at the proper stage of differentiation. Ironically, CD147 was tested as the cell surface marker for cone enrichment. CD147 is encoded by the basigin (*BSG*) gene, which also encodes for the splice variant BSG1, the cell-surface receptor of rod-derived cone viability factor (RdCVF) [53]. For advanced stages of retinitis pigmentosa, since cone survival depends on the rods, it is advisable to recapitulate rod to cone signaling by RdCVF upregulation [54]. Recent demonstration of an indirect mechanism of cone rescue after transplantation of rod precursors in a pig model of retinitis pigmentosa seems to act according to that scenario since rods, grafted in the subretinal space of the pig retina, restore glucose transport and promote the regrowth of cone outer segments [55]. 

Transplanted cone precursors exchange cytoplasmic material with host photoreceptors, both rods and cones in the wild-type mouse retina. Therefore, the phenomenon is not cell-restricted by selective properties of rods [40]. The transplanted cone precursors show that the signal arising from the ONL of the host results in cytoplasmic material exchanges [56]. Preliminary evidence shows that the phenomenon is shared among many species including primates, for which submacular injection of photoreceptor precursors results in signal in the ONL of the host squirrel monkey (*Saimiri sciureus*) retina [57]. Transplantation favors the most abundant and accessible cell type present in the host retina in the absence of photoreceptor degeneration. Cytoplasmic material exchange is bi-directional and occurs between host photoreceptors and transplanted photoreceptor precursors, and vice versa, but the transfer is a developmentally regulated phenomenon, dependent on the developmental stage of the transplanted cells, that appears to be restricted to immature photoreceptor precursor cells [40,56]. The extent to which cytoplasmic material exchange occurs could question the current model of differentiation of retinal cells during development, for which a significant proportion of the results sustaining the understanding of retinal cell fate from post-mitotic retinal precursor cells was obtained using reporter genes [23,58]. An inherent leakiness of the cellular compartmentalization by plasma membrane permitting the phenomenon would impair lateral inhibition. The delta-Notch signaling relies on the fact that two neighboring cells express a non-secreted ligand and its cell-surface receptor. The ligand produced by the donor cell, by binding to the receptor of the recipient cell, triggers an intracellular cascade of events leading to a reduced ligand expression by the recipient cell, finally leading to cell-fate specification. Leaky cytoplasmic material exchanges between these two cells would eventually antagonize this differentiation process. However, experimental evidence supporting an instructive role of cytoplasmic material exchanges in cell-fate decision during retinal development has presently not been reported.

### 2.3. What Did We Learn?

The observation of cytoplasmic material exchange does not rule out the possibility that, under certain circumstances, transplanted photoreceptors could make synapses with adult host retinal cells, though this is currently not widely accepted [24]. The fact that cytoplasmic material exchange is regulated by the development stage of the transplanted cells argues for mechanisms underlying genetic control. The mechanism of cell material fusion remains to be elucidated, but most studies highlight its potential role as a novel therapeutic approach to restore cone function in models of cone dystrophies. The concept of cytoplasmic exchange is emerging from cell transplantation studies as a novel direction for cell-based therapies [40]. Cytoplasmic material exchange can theoretically result from communications through tunneling nanotubes or by vesicular transport processes that are both independent of soluble factors [59,60].

Structurally, tunneling nanotubes are membrane protrusions supported by a backbone of actin polymer that could be identified between transplanted photoreceptor precursors and photoreceptors in the ONL of the host retina by immunostaining and scanning electron microscopy [61]. Such structures have yet to be observed. Alternatively, the release of extracellular vesicles by transplanted photoreceptor precursors and the release of their contents to host photoreceptors, eliciting functional rescue, deserve to be studied as a possible alternative mechanism. Among the diversity of the extracellular vesicles, exosomes (40–150 nm) and microvesicles (0.1–1 mm) represent the highest potential for mediating cytoplasmic material exchange between cells after photoreceptor transplantation [62]. Exosomes are extracellular vesicles formed after inward budding of the plasma membrane of intracellular endosomes and are released by all cell types. Extracellular exosomes can fuse with the plasma membranes of another cell to deliver their contents into its cytoplasm. The outcome of this cell-to-cell communication depends on the nature of the cargoes. Exosomes contain lipids, proteins, and nucleic acids. Contents include various proteins involved in membrane transport and fusion, as Rab GTPases and annexins, but also proteins highly expressed in the cell of origin, as GFP by transplanted photoreceptor precursors. It is now possible to address the importance of exosomes in cytoplasmic material exchange after photoreceptor transplantation by silencing the genes coding for proteins involved in membrane transport and fusion in photoreceptor precursors. The half-life of GFP has a direct implication in the biochemical nature of the exchanged material. It was noticed that host photoreceptors would require a near-constant supply of GFP mRNA or protein to be detected, in accordance to what has been observed [56]. It is necessary to maintain the presence of donor cells to be able to observe the transfer of GFP; the donor cell nuclei remain in the subretinal space where they were grafted, while donor-derived cytoplasm is transferred into host photoreceptors. Stable and prolonged contacts are reminiscent of the transfer of disks of photoreceptor outer segments to RPE cells by phagocytosis. The transplantation of photoreceptor precursors isolated from mice carrying a disruption of genes involved in photoreceptor outer segment renewal, such as mice that carry a null allele of the GTPase *Rab28* gene that cause recessive cone-rod dystrophy, would allow for an investigation of any correlation between the two processes [63]. Extracellular vesicles carry mRNA and miRNA, and are able to modulate gene expression in recipient cells [64]. The transplantation of rodents with a silencing RNA (siRNA) targeting GFP mRNA in host photoreceptors should address the contribution of GFP mRNA in cytoplasmic material exchange from transplanted photoreceptor precursors expressing that reporter. If the RNA is the cargo exchanged, RNA interference will reduce GFP expression by the host photoreceptor cells.

Broadly, these studies showed that cytoplasmic material exchange has the potential to lead to novel therapeutic approaches. For example, photoreceptor precursors can be engineered to produce in vitro exosomes or microvesicles loaded with therapeutic mRNAs and/or proteins to correct the gene defect in photoreceptors of the host retina [65]. This may overcome a limit encountered in corrective gene therapies using recombinant AAV vectors by possibly allowing simultaneous delivery of several genes. From all ongoing studies will certainly emerge a new field of gene therapy aiming at restoring gene function by delivering mRNAs instead of genes. This messenger RNA therapy relying on cytoplasmic material exchanges is an unsuspected development of photoreceptor transplantation that may finally be exploited. 

## 3. Transplantation of RPE Cells in Retinal Therapy

### 3.1. Genetic Considerations

Following previous reviews [2,66,67], the current review will address RPE transplantation through the angle of its specific aspects as compared to photoreceptor transplantation. The main differences concern genetic and cell biology concepts that rationalize the use of RPE transplantation. The field of photoreceptor transplantation is dominated by studies of the benefit of allographs and autographs preceded by gene-editing to treat the Mendelian forms of inherited retinal degenerations. RPE transplantation mostly, but not exclusively, targets AMD, a multifactorial disease [68,69]. AMD is the leading cause of blindness with 196 million patients projected to suffer from this disease worldwide by 2020. GWAS have identified 34 loci carrying alleles conferring a risk to develop AMD [11,12]. The rationale in these studies is the existence of linkage disequilibrium between common genetic markers, most often an allele of a single nucleotide polymorphism (SNP), and a causative allele located in the chromosomal vicinity [70]. This has a profound consequence in the field; not only does AMD result from the interaction of multiple causing alleles with environmental factors during aging, but the causative alleles are most often unknown. In terms of allografts, the most reasonable approach would be to transplant RPE cells or iPS-derived RPE cells that do not carry the risks alleles at the 34 identified loci. Unfortunately, people with this genotype are extremely rare. Out of 3272 AMD patients and controls genotyped from a Paris cohort, none has that non-risked genotype, and the question of the donor remains open and hints that the ideal donors are extremely rare. Each of these individual risk alleles does not exert an even influence on the occurrence of AMD. The grading system relies on the odds ratio (OR), which is defined as the ratio of two odds, the odds of the occurrence of AMD for individuals carrying a given risk allele and the odds of the occurrence of AMD for individuals not carrying that allele. Starting from 1, according to that scale, the two major AMD risk alleles, in the complement factor H (*CFH*) and *ARMS2/HTRA1* genes, are 2.1 and 2.8, respectively. The OR from the lactate transporter gene *SLC16A8* is 1.13 [11]. One should here keep in mind that OR is calculated from allele frequency, which depends on the polymorphic information content, the highest corresponding to the maximum heterozygous individuals following the Hardy–Weinberg equilibrium. That is to say that, depending on the genetics of the studied population, OR may or may not be the exact measure of the risk to develop the disease. Allografts would theoretically benefit from gene editing of the major causal alleles, such as the CFH^402H^ coding allele, but correcting other alleles associated with AMD will not help, given that the involvement is indirect through linkage disequilibrium with an uncorrected and unknown causal allele.

Even if the disease results from dysfunction of foveal cone photoreceptors at the center of the macula, RPE is considered by many biologists and clinicians to be a major player in AMD [19]. With aging, the elimination of photoreceptor disks by RPE becomes increasingly inefficient, leading to progressive accumulation of deposits (drusen) at the basal membrane of the RPE resembling atherosclerotic plaques. The presence of large drusen represents an early phase of the disease and predicts severe clinical outcome as choroid neovascularization (wet AMD) or geographic atrophy (dry AMD). Choroid neovascularization (CNV) occurs by the growth of new blood vessels leading to rapid loss of vision. CNV constitutes only 10–15% of all AMD cases that are currently treated by anti-VEGF therapy. Geographic atrophy is a progressive development of focal loss of RPE and photoreceptors leading to severe visual impairment. Animal models of AMD will be invaluable tools, but the affected region of the retina, the macula, is not present in non-primate species, so a rodent model of AMD is still missing. The lack of a proper animal model led researchers in early RPE transplantation studies to use the Royal College of Surgeons (RCS) rat, a recessive model of retinitis pigmentosa with a loss of function in the *Mertk* gene encoding the receptor tyrosine kinase involved in photoreceptor outer segment phagocytosis by the RPE [71,72]. From initial transplantations of RPE cell lines, progress has been achieved until today with differentiated human iPS-RPE cells [73,74,75,76]. Subretinal transplantation of hESC-derived RPE cells can also rescue photoreceptors and prevent visual loss in preclinical models of macular degeneration, including non-rodent species [77,78].

These animal studies are proof of concept for human clinical trials [79,15]. RPE transplantation has to overcome the immune rejection despite the existence of ocular immune privilege. Inflammatory cell invasion, T cells, retinal microglia, and antigen-presenting cells are found in the retina, subretinal space, and choroid after RPE transplantation [80]. Those are strong arguments for the use of RPE made from iPSCs originating from the recipient patient.

### 3.2. Mode of Action

The possible lack of complete differentiation to RPE cell-fate of the transplanted iPSC is of concern since RPE cells are not post-mitotic and could grow in situ, resulting in tissue damage [81]. Considering that the RPE is an epithelium, RPE derived from iPSCs (iPS-RPE) can be transplanted as isolated cells but also as cell sheets using several technical devices as human amniotic membrane [76,82]. The morphology of these cells is similar to mature RPE cells, and molecular analyses have shown that they express numerous RPE markers. They are capable of phagocytosing photoreceptor material, in vitro and in vivo, following transplantation into the RCS rat [76]. The pigmentation of the iPS-RPE is considered as a hallmark for the state of maturation. In most reported studies, iPS-RPE cells are grown in vitro to confluence before being transplanted. This cell expansion can result in the dedifferentiation of the iPS-RPE cells [83]. The homeoprotein OTX2 is involved in the expression of several RPE markers and its expression declines during the expansion period. Using *Otx2*-genetically modified primary RPE cells, OTX2 was reported to increase the rescue of photoreceptors after transplantation.

RPE cells are essential for phagocytosing and recycling photoreceptor outer segments but also regenerating photoreceptor visual pigment through the vitamin A cycle. They are also essential to the regulation of ionic and metabolic fluids. They form the blood-outer retinal barrier. Which of these biological activities restored by RPE transplantation are necessary to prevent photoreceptor degeneration? iPS-RPE cells grown in vitro are able to phagocytize photoreceptor outer segments but this capacity decreases with time in culture [81]. Transplanted iPS-RPE generates a protection of photoreceptors of the host retina well outside the site where the graft is located, which suggests that the protective activity is at least partly non-cell autonomous. iPS-RPE cells grown in vitro produce the visual chromophore 11-*cis*-retinal. After transplantation in the retina of mice carrying mutations in genes essential for vitamin A cycle, there is an improvement in vision [84,85]. Cones can also use an alternative recycling pathway for which Müller cells isomerize all-*trans*-retinol to 11-*cis*-retinol, which is then oxidized by cones to 11-*cis*-retinal for pigment regeneration [86]. While the restoration of the vitamin A cycle by RPE transplantation is certainly the mechanism by which vitamin A cycle mutants are protected, it is possible that the protection of cone function in other models with photoreceptor degeneration does result from the preservation of rods via rod to cone signaling [83,87].

The non-cell autonomous property of the protective activity led to the concept of trophic factors secreted by the transplanted RPE cells [88]. Pigmented epithelium-derived factor (PEDF/SERPINF1), originally identified in the conditioned medium of cultured human fetal RPE cells, is secreted on the apical side of the RPE, toward photoreceptors, and protects them by decreasing their intracellular calcium and apoptotic signaling [89]. PEDF/SERPINF1 protects photoreceptors by binding to a PEDF cell-surface receptor, the patatinlike phospholipase domain-containing 2 (PNPLA2) [90]. The *Serpinf1^−/−^* mouse displays retinal abnormalities but its phenotype recapitulates mainly the hypomineralization and low bone mass phenotypes observed in patients with osteogenesis imperfecta, type VI [91,92,93]. 

The therapeutic potential of PEDF/SERPINF1 is not supported by a private local and specific interaction between RPE and photoreceptors, as reported for the RdCVF metabolic and redox signaling in the retina [94]. Its contribution to the beneficial effect of RPE transplantation on photoreceptor survival remains to be demonstrated by transplanting iPS-RPE cells carrying a deletion of the *PEDF/SERPINF1* gene [95]. The expression of the *NXNL1* gene, encoding for RdCVF, is decreased following retinal detachment, which leads to a lack of trophic support for cones. Contrarily, expression of the *PEDF/SERPINF1* mRNA is increased, suggesting the trophic support for cones in retinal detachments [96].

Alternatively, non-cell autonomous effects can result from non-peptidic molecules. After RPE transplantation in the RCS rat, immunostaining for Na/K-ATPase was observed in the photoreceptor inner segments, specifically in grafted regions as in control retinas [97]. Other studies highlighted the role of the Na/K-ATPase in photoreceptor function and survival, as the mutations in the retinoschisin (*RS1*) gene cause X-linked juvenile retinoschisis, a hereditary retinal dystrophy [98]. Indeed, retinoschisin (RS1) interacts with Na/K-ATPase through the ATP1B2 subunit of [99]. In the retinas of *Atp1b2^−/−^* mice, photoreceptors undergo increasing apoptosis from Post-Natal Day 9 [100]. Thus, insufficient Na/K-ATPase activity may contribute to the progressive photoreceptor cell death observed in *Rs1^−/−^* mice. Recessive mutations in the *KCNJ13* gene encoding for a potassium transporter cause Leber congenital amaurosis, a severe form of inherited retinal degeneration [101]. RPE-specific inactivation of the *Kcnj13* gene in the mouse causes photoreceptor degeneration [102]. KCNJ13 is located in the apical membrane of the RPE, toward photoreceptors, where it controls potassium homeostasis in the microenvironment surrounding photoreceptors, an essential role for photoreceptor function. Na/K-ATPase pumps potassium from the extracellular space to maintain a voltage gradient across the membrane of the cell, a mechanism necessary for photoreceptor function. It has been shown that OTX2 upregulates 9-fold the expression of *KCNJ13* in RPE cells by binding to an element in its promoter [83], so that the enhanced and diffused protection of photoreceptors by transplantation of *Otx2*-genetically modified primary RPE cells could theoretically be mediated through a partial restoration of potassium homeostasis in the retina.

In addition, Na/K-ATPase activity is regulated by ATP preferentially produced by glycolysis and not by oxidative phosphorylation [103]. Aerobic glycolysis is a hallmark of photoreceptors [53,104]. Aerobic glycolysis produces ATP and lactate that is transported to the choroid vasculature through the RPE via two distinct lactate transporters, MCT1 (*SLC16A1*) in the apical side toward photoreceptors and MCT3 (*SLC16A8*) on the basal side [105]. The disruption of the *Slc16a8* gene that is exclusively expressed by the RPE leads to an excess of lactate in the outer retina of mice, at the level of photoreceptors that impairs their function [106]. In human, the *SLC16A8* gene carries risk alleles and a putative causative allele for AMD [11,12]. It has been shown that OTX2 upregulates 2.5-fold the expression of *SLC16A8* in RPE cells [83]. Following previous reasoning, a partial restoration of lactate transport through the RPE may also contribute to the enhanced protection of photoreceptors by transplantation of *Otx2*-genetically modified primary RPE. The contribution of these hypothetical non-cell autonomous mechanisms in the protection of photoreceptors in the animal could be studied in the future by transplanting iPSCs edited using CRISPR-Cas9 technology, by deleting the genes encoding for MCT3 and KCNJ13 in the cells to be transplanted [22].

To achieve a successful transfer of retinal cell transplantation into clinical practice, some key elements are to be taken into consideration: medically, the clinical criteria that define which patients can enroll; technically, the source of the cells to be transplanted and the risk of any side effects, such as tumor formation and graft rejection by the host immune system [107]. There are currently 18 clinical trials reaching Phase II, in which the benefit of cell transplantation is measured, using either photoreceptor or RPE cells: 5 for Mendelian and polygenic retinal diseases; 3 for retinitis pigmentosa; and 10 for AMD (https://clinicaltrials.gov). Ongoing study results will determine the future of such an approach in the field of retinal diseases. The results of randomized clinical trials that test the safety and efficacy of retinal cell transplantation will have to be precisely weighted, taking into account their benefit as compared to the inherent risks, suggesting that care should be taken when expanding such treatments to a clinical setting [108]. 

## 4. Concluding Remarks

Taking together the current status of cell-based therapies for retinal diseases, one could perceive that this field has been dominated by a result-oriented culture stimulated by the enthusiasm of its application in medicine. This approach has produced enormously successful studies and highlighted many questions on the molecular and cellular mechanisms involved in the protection and the restoration of vision. While some of these approaches are currently being transferred to the clinic, the way the benefit will be obtained for the patient has not been elucidated in detail yet. Progress made in the generation of iPSCs for producing cells to be transplanted as well as the progress of the gene editing of these cells and the animal host will contribute to the field’s understanding and most likely pave the way for novel therapeutic approaches for retinal degenerations.
